# Innate Immune Response of Human Alveolar Macrophages during Influenza A Infection

**DOI:** 10.1371/journal.pone.0029879

**Published:** 2012-03-02

**Authors:** Jieru Wang, Mrinalini P. Nikrad, Emily A. Travanty, Bin Zhou, Tzulip Phang, Bifeng Gao, Taylor Alford, Yoko Ito, Piruz Nahreini, Kevan Hartshorn, David Wentworth, Charles A. Dinarello, Robert J. Mason

**Affiliations:** 1 Department of Medicine, National Jewish Health, Denver, Colorado, United States of America; 2 J. Craig Venter Institute, Rockville, Maryland, United States of America; 3 School of Medicine Cancer Center, University of Colorado at Denver and Health Sciences Center, Aurora, Colorado, United States of America; 4 Department of Oncology, School of Medicine, Boston University, Boston, Massachusetts, United States of America; 5 Division of Infectious Diseases, University of Colorado at Denver and Health Sciences Center, Aurora, Colorado, United States of America; 6 Department of Medicine, School of Medicine, University of Colorado at Denver and Health Sciences Center, Aurora, Colorado, United States of America; Louisiana State University, United States of America

## Abstract

Alveolar macrophages (AM) are one of the key cell types for initiating inflammatory and immune responses to influenza virus in the lung. However, the genome-wide changes in response to influenza infection in AM have not been defined. We performed gene profiling of human AM in response to H1N1 influenza A virus PR/8 using Affymetrix HG-U133 Plus 2.0 chips and verified the changes at both mRNA and protein levels by real-time RT-PCR and ELISA. We confirmed the response with a contemporary H3N2 influenza virus A/New York/238/2005 (NY/238). To understand the local cellular response, we also evaluated the impact of paracrine factors on virus-induced chemokine and cytokine secretion. In addition, we investigated the changes in the expression of macrophage receptors and uptake of pathogens after PR/8 infection. Although macrophages fail to release a large amount of infectious virus, we observed a robust induction of type I and type III interferons and several cytokines and chemokines following influenza infection. CXCL9, 10, and 11 were the most highly induced chemokines by influenza infection. UV-inactivation abolished virus-induced cytokine and chemokine response, with the exception of CXCL10. The contemporary influenza virus NY/238 infection of AM induced a similar response as PR/8. Inhibition of TNF and/or IL-1β activity significantly decreased the secretion of the proinflammatory chemokines CCL5 and CXCL8 by over 50%. PR/8 infection also significantly decreased mRNA levels of macrophage receptors including C-type lectin domain family 7 member A (CLEC7A), macrophage scavenger receptor 1 (MSR1), and CD36, and reduced uptake of zymosan. In conclusion, influenza infection induced an extensive proinflammatory response in human AM. Targeting local components of innate immune response might provide a strategy for controlling influenza A infection-induced proinflammatory response *in vivo*.

## Introduction

Alveolar macrophages (AM) reside at the air-tissue interface in the lung and are one of the first lines of defense that interact with inhaled microorganisms and particles [1]. They play a critical role in homeostasis, host defense, and tissue remodeling [2], and they are readily infected by influenza [3]. AM express many pattern recognition receptors (PRRs) to help recognize the pathogen-associated molecular patterns (PAMPs) on the surface of microorganisms [4], [5]. They are important in initiating response to influenza, regulating the inflammatory response, and potentially limiting secondary bacterial infections [6].

Influenza A virus causes seasonal and pandemic flu, both of which pose significant public health burdens. Influenza viral antigens have been detected in AM from humans and many animal species [7]–[15], and AM are critical for controlling viral replication *in vivo* [11], [14]. Recently, several groups have explored the responses of human monocyte-derived macrophages to avian and/or seasonal flu viral infection using a genome-wide approach [16]–[18]. Avian H5N1 and human H1N1 and H3N2 viruses induce increases in similar groups of genes despite the stronger response induced by pathogenic avian viruses compared to seasonal flu viruses in human monocyte-derived macrophages [16]–[19]. However, the genome-wide response of resident human AM to influenza infection has not been reported. Our previous study showed that cultured primary human AM support a productive infection with H5N1 but not H1N1 and H3N2 influenza viruses though AM express both avian and human influenza receptors [3], [19]. However, human monocyte-derived macrophages support productive infection with both human and avian viruses [19]–[21]. These results suggest that the response of human AM to influenza might be different from the response of human macrophages derived from peripheral blood [22].

The purpose of our study was to use a genome-wide approach to define the innate immune response of human AM to influenza. Using H1N1 influenza virus PR/8, we performed gene profiling of virus-infected human AM at 4 and 24 h post inoculation (hpi) and verified the alterations in IFN-related genes by real-time RT-PCR and cytokine response by ELISA. We investigated the kinetics of infection-induced cytokine response in human primary AM infected with both live and UV-inactivated PR/8 and the contemporary H3N2 virus A/New York/238/2005 (NY/238) [23]. We also determined if the cytokine response was amplified by paracrine proinflammatory cytokines, TNF-α and IL-1β. In addition, we explored whether influenza infection diminishes gene expression of macrophage scavenger receptors, which could contribute to the impaired ability of AM to clear other pathogens after influenza.

## Results

### Overview of global gene expression altered by influenza infection

Viral infection resulted in significant alterations of mRNA levels in 1,347 transcripts at 4 hpi and 2,152 transcripts at 24 hpi; these transcripts mapped to 1,077 (4 hpi) and 1,493 (24 hpi) known genes. Tables 1 and 2 show the top 25 genes that were up-regulated or down-regulated by influenza virus. The complete list of altered genes is listed in Data S1. To identify the cellular functions and pathways affected by the infection, the array data were processed by Ingenuity Pathway Analysis (IPA) using IPA version 8.0 (Ingenuity® Systems, Redwood City, CA), which associates differentially regulated genes with known specific biological pathways based on information from published literature (www.ingenuity.com). The results from IPA indicate some functional groups of genes were changed at both time points. These genes are involved in antimicrobial and inflammatory responses, cell death, cancer, infection mechanisms, cellular growth and proliferation, cell-mediated immune responses, and immune cell trafficking. Interferon regulatory factor (IRF) activation and PRR signaling were the most prominent pathways activated by viral infection at both time points. In addition, retinoic acid-induced gene-1 (RIG-I) and interferon (IFN) signaling were dominant at 4 hpi, whereas homeostasis-related pathways such as IL-10 and IL-6 were activated at 24 hpi.

**Table 1:** The top 25 genes up-regulated or down-regulated by PR/8 infection in human AM at 4 hpi.

Up-regulated			Down-regulated		
Gene name	Symbol	Fold	Gene name	Symbol	Fold
interferon, beta 1, fibroblast	IFNB1	6940	ceroid-lipofuscinosis, neuronal 8	CLN8*	73
interferon stimulated exonuclease gene 20 kDa	ISG20	1916	plasminogen activator, urokinase	PLAU*	23
interferon, alpha 8	IFNA8	978	Kruppel-like factor 13	KLF13*	19
chemokine (C-X-C motif) ligand 11	CXCL11	850	metastasis associated in colon cancer 1	MACC1	19
interferon, alpha 21	IFNA21	737	DNA fragmentation factor, 45 kDa, alpha polypeptide	DFFA	16
interferon, alpha 13	IFNA13	642	dual-specificity tyrosine-(Y)-phosphorylation regulated kinase 2	DYRK2*	16
interferon, alpha 4	IFNA4	602	sorting nexin 12	SNX12	15
interferon, alpha 1	IFNA1	502	zyg-11 homolog B (C. elegans)	ZYG11B	15
leprecan-like 1	LEPREL1	370	MAX binding protein	MNT	14
interferon, alpha 7	IFNA7	317	G protein-coupled receptor 157	GPR157	14
matrilin 1, cartilage matrix protein	MATN1	316	X-linked inhibitor of apoptosis	XIAP*	14
lactate dehydrogenase A-like 6B	LDHAL6B	301	baculoviral IAP repeat-containing 4	BIRC4*	14
BCL2-like 14 (apoptosis facilitator)	BCL2L14	286	potassium voltage-gated channel, Isk-related family, member 3	KCNE3	14
Interleukin 29	IL 29	252	slingshot homolog 1 (Drosophila)	SSH1*	13
similar to Immune-responsive protein 1	LOC730249	224	tubulin tyrosine ligase-like family, member 4	TTLL4	13
chemokine (C-C motif) ligand 5	CCL5	223	Hypothetical protein LOC158402	LOC158402	13
glucagon	GCG	218	transforming growth factor, beta receptor 1	TGFBR1	12
hairy and enhancer of split 4 (Drosophila)	HES4	189	SRY (sex determining region Y)-box 4	SOX4*	11
	DKFZp434A119	168	tribbles homolog 3 (Drosophila)	TRIB3	11
tumor necrosis factor (ligand) superfamily, member 10	TNFSF10	126	pleckstrin homology-like domain, family A, member 1	PHLDA1*	11
fibroblast activation protein, alpha	FAP	125	peptidylprolyl isomerase F (cyclophilin F)	PPIF*	10
interferon, alpha 17	IFNA17	122	hypothetical protein LOC153346	LOC153346	10
indoleamine-pyrrole 2,3 dioxygenase	INDO	121	ankyrin repeat domain 50	ANKRD50	10
HESX homeobox 1	HESX1	92	calmodulin regulated spectrin-associated protein 1	CAMSAP1*	10
chemokine (C-X-C motif) ligand 9	CXCL9	91	methyltransferase 10 domain containing	METT10D	10

Human AM from 3 non-smoking donors were isolated, cultured, and infected by PR/8 virus at a MOI of 0.5. The gene profiling of infected and non-infected cells at 4 hpi from each donor was examined by microarray experiments using Affymetrix HG-U133 Plus 2.0 chips (Affymetrix, Santa Clara, CA). The filtered gene list was generated as described in the Section of Methods. The data show the top 25 genes up-regulated or down-regulated altered by viral infection.

*indicates similar results from multiple probes.

**Table 2:** The top 25 genes up-regulated or down-regulated by PR/8 infection in human AM at 24 hpi.

Up-regulated			Down-regulated		
Gene name	Symbol	Fold	Gene name	Symbol	Fold
chemokine (C-X-C motif) ligand 10	CXCL10	2900	C-type lectin domain family 7, member A	CLEC7A*	122
tissue factor pathway inhibitor 2	TFPI2	1003	lysophospholipase-like 1	LYPLAL1	63
chemokine (C-X-C motif) ligand 11	CXCL11	987	metastasis associated in colon cancer 1	MACC1	63
chromosome 9 open reading frame 152	C9ORF152	444	progestin and adipoQ receptor family member V	PAQR5	58
fibroblast activation protein, alpha	FAP	384	calcium channel, voltage-dependent, L type, alpha 1D subunit	CACNA1D	35
hairy and enhancer of split 4 (Drosophila)	HES4	368	membrane-associated ring finger (C3HC4)*	MARCH1	34
interferon induced transmembrane protein 1	IFITM1*	353	solute carrier organic anion transporter family, member 2B1	SLCO2B1*	33
	DKFZp434A119	339	transmembrane 7 superfamily member 4	TM7SF4	29
synaptopodin 2	SYNPO2	334	inositol(myo)-1(or 4)-monophosphatase 2	IMPA2	29
chemokine (C-X-C motif) ligand 9	CXCL9	305	tumor necrosis factor (ligand) superfamily, member 12	TNFSF12	28
Interlukin 27	IL27	299	cathepsin S	CTSS	27
ATPase, Class I, type 8B, member 2	ATP8B2	236	macrophage scavenger receptor 1	MSR1*	25
apolipoprotein B mRNA editing enzyme, catalytic polypeptide-like 3B	APOBEC3B	230	mitochondrial antiviral signaling protein	MAVS*	25
interleukin 28A (interferon, lambda 2)	IL-28A	227	prostaglandin F2 receptor negative regulator	PTGFRN	25
chemokine (C-C motif) ligand 5	CCL5	225	solute carrier family 46, member 3	SLC46A3	23
BCL2-like 14 (apoptosis facilitator)	BCL2L14	195	thioesterase superfamily member 2	THEM2	23
bone morphogenetic protein 2	BMP2	191	hydroxyprostaglandin dehydrogenase 15-(NAD)	HPGD*	23
transmembrane protein 47	TREM47	170	hexokinase 3 (white cell)	HK3	21
somatostatin receptor 2	SSTR2	169	choline dehydrogenase	CDH	21
interferon, alpha 1	IFNA1	167	lung cancer metastasis-associated protein	NAG1	20
interleukin 29 (interferon, lambda 1)	IL-29	135	ribulose-5-phosphate-3-epimerase	RPE	19
interferon induced transmembrane protein 1 (9–27)	IFITM1	133	MPN domain containing	MPND	18
interferon stimulated exonuclease gene 20 kDa	ISG20	108	C-type lectin domain family 4, member A	CLEC4A*	18
guanylate binding protein 1, interferon-inducible, 67 kDa	GBP1	95	deoxyribonuclease II beta	DNASE2B	18
similar to Immune-responsive protein 1	LOC730249	91	macrophage expressed gene 1	MPEG1*	17

Human AM from 3 non-smoking donors were isolated, cultured, and infected by PR/8 virus at a MOI of 0.5. The gene profiling of infected and non-infected cells at 24 hpi from each donor was examined by microarray experiments using Affymetrix HG-U133 Plus 2.0 chips (Affymetrix, Santa Clara, CA). The filtered gene list was generated as described in the Section of Methods. The data show the top 25 genes up-regulated or down-regulated altered by viral infection.

*indicates similar results from multiple probes.

### Influenza infection triggers an early and strong response of IFN signaling

**Figure 1:**
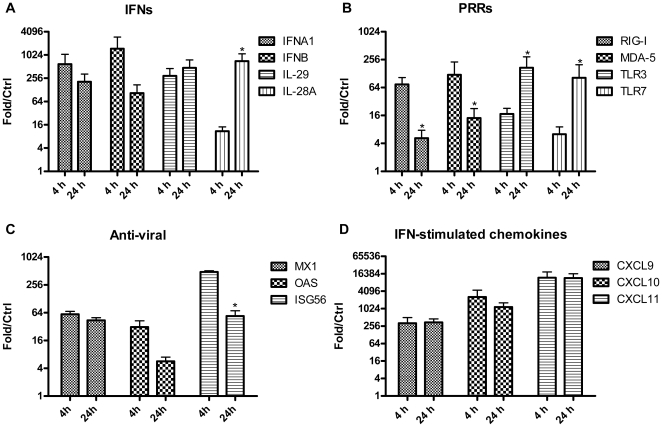
Verification of virus-induced increase of mRNAs of IFN and IFN-associated genes by quantitative RT-PCR. Human AM isolated from donor lungs were cultured and infected with PR/8 at a MOI of 0.5. Total RNA was isolated at 4 and 24 hpi from virus-infected and non-infected cultures. mRNA expression of IFN and IFN-associated genes were measured by real-time RT-PCR. The data represent mean+SE of the relative expression levels of each gene in infected cultures to that of non-infected controls after normalization to the level of the constitutive probe cyclophilin B, N = 8. Expression of all the tested genes are significantly different between virus-infected cultures and non-infected cultures, * indicates that the difference between 4 and 24 hpi is statistically significant (P<0.05).

As shown in Table 1 at 4 hpi, seven out of the top ten PR/8 up-regulated genes were type I IFN family members, and another two up-regulated genes, IFN-stimulated gene (ISG) 20 and CXCL11, were IFN-stimulated genes [24]. At 24 hpi, IFN-stimulated genes CXCL9–11, and IFITM1 were among the top ten genes up-regulated by PR/8 (Table 2). Therefore, we verified the microarray data with a focus on IFN-associated genes by real-time RT-PCR (Figure 1). As shown in Figure 1A, PR/8 infection induces an early response in type I IFN genes IFNA1 and IFNB as well as type III IFN genes IL-29 and IL-28A, although the degree of increase was slightly less than that of most type I IFN genes (Tables 1 and 2). Along with the increased IFN gene expression, the infection also increased expression of well-known PRR genes associated with IFN production. mRNA levels of RIG-I and melanoma differentiation associated protein-5 (MDA-5) were mainly increased at 4 hpi, whereas TLR3 and 7 were mainly stimulated at 24 hpi (Figure 1B). The infection also significantly increased mRNA of IFN-stimulated anti-viral genes myxovirus (influenza virus) resistance 1 (MX1), 2′5′ oligoadenylate synthase (OAS), and IFN-stimulated gene 56 (ISG56) (Figure 1C and Data S1), as compared to control cells.

### Influenza infection induces an extensive cytokine and chemokine response

In addition to IFN related genes, PR/8 significantly increased the expression of many cytokine genes and cytokine-regulated genes. These included the proinflammatory cytokines TNF-α (7.3-fold at 4 hpi and 25-fold at 24 hpi), IL-1α (6.9-fold at 24 hpi), and IL-1β (2.3-fold at 4 hpi and 16.3-fold at 24 hpi). We verified the alteration in IL-1α and IL-1β by real-time RT-PCR (data not shown). The infection also upregulated expression of TNF-α induced proteins 2, 3, 6, and 8, as well as TNF receptor family members 9 and 10. Expression of IL-1 family members interleukin 1 receptor 1 (IL-1R1) and the IL-1 receptor antagonist (IL-1Ra) was also increased (Data S1). In addition, PR/8 infection up-regulated mRNA expression of many chemokine genes including CC chemokines CCL2–5, and CCL20, as well as CXC chemokines CXCL9–11 (Data S1 and Tables 1 and 2). CXCL9–11 were markedly increased when compared to controls both in the microarray studies and in additional verification studies (Tables 1 and 2 and Figure 1D). CCL5 was the most increased CC chemokine (232-fold at 4 hpi, 234-fold at 24 hpi) (Tables 1 and 2).

Besides the increased mRNA expression of proinflammatory mediators, PR/8 also increased mRNA expression of the anti-inflammatory cytokine IL-10 (1.9-fold at 4 hpi and 2.8-fold at 24 hpi), its receptor (8-fold at 4 hpi and 6.8-fold at 24 hpi), and suppressor of cytokine signaling (SOCS)1 (9.4-fold at 4 hpi and 12.1-fold at 24 hpi) and SOCS3 (5.4-fold at 4 hpi), which have been shown to be important in turning off inflammatory responses and dampening a robust innate immune response [25]. PR/8 also upregulated expression of IL-6 (36.9-fold at 4 hpi and 66.6-fold at 24 hpi), another important cytokine responsible for homeostasis, and several cytokines that activate and regulate adaptive immune response, especially IL-15 and its receptor, IL-23A, and IL-27 [26]–[28] (Data S1).

**Figure 2:**
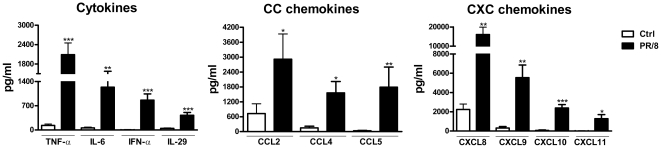
Verification of virus-induced secretion of chemokines and cytokines by ELISA. Human AM isolated from donor lungs were cultured and infected by PR/8 at a MOI of 0.5. Secretion of chemokines and cytokines from infected and non-infected cultures was measured by ELISA at 24 hpi. The data represent mean+SE of each released cytokine and chemokine (pg/ml). The number of individual donors ranged from 8 to 16. * indicates P<0.05, ** indicates P<0.01, *** indicates P<0.001 vs. non-infected cells.

We verified the putative increases in secreted cytokines and chemokines at the protein level by ELISA in 8–14 additional donors. As shown in Figure 2, PR/8 infection significantly increased secretion of cytokines of TNF-α, IL-6, IFN-α, and IL-29, and CXC chemokines CXCL8–11 as well as CC chemokines CCL2, 4 and 5. Consistent with the mRNA data, CXCL10, CXCL11 and CCL5 were the main chemokines induced by the virus, and AM secreted slightly more IFN-α than IL-29 (Figure 2).

### Secretion of cytokines and chemokines occurs without the release of a significant amount of infectious viral particles

**Figure 3:**
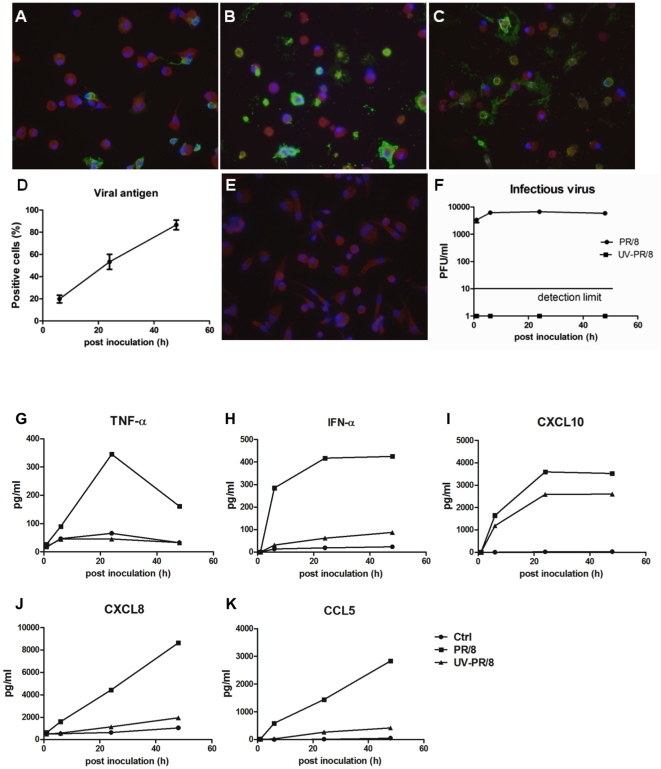
Kinetics of influenza infection with live and UV-inactivated PR/8. Primary AM were cultured and infected by live PR/8 at a MOI of 0.5 or the equal amount of UV-inactivated PR/8, and cells were harvested at designated time post inoculation. Panels A–F. Kinetics of viral antigen synthesis and infectious virus release. Panels A–C show representative immunofluorescence staining for influenza HA from live PR/8-infected AM culture at 6, 24, and 48 hpi. Panel D shows the quantitation of these experiments. The data represent mean±SE of percentage of positive-stained cells from 6 donors. Panel E. Representative staining of viral antigen in UV-inactivated PR/8 infection at 48 hpi. Panel F. Representative release of infectious viral particles from both live and UV-inactivated PR/8-infected AM from 6 donors. Panels G–K. Time course of cytokine and chemokine response in PR/8-infected AM. The supernatant from cultured cells were collected at 1, 6, 24, and 48 hpi. Secretion of TNF-α (Panel G), IFN-α (Panel H), CXCL10 (Panel I), CXCL8 (Panel J), and CCL5 (Panel K) was measured by ELISA. Data show representative release of each cytokine from infected AM of 6 donors that all showed similar response.

From our previous studies we knew that AM do not release a significant amount of infectious virus particles after infection with human influenza viruses [3], [19]. To investigate whether the infected macrophages were synthesizing viral proteins, we performed a time-course infection experiment in AM from an additional 4 donors using both live (Figure 3A–D) and UV-inactivated PR/8 viruses (Figure 3E), examined the kinetics of viral antigen synthesis by staining hemagglutinin (HA) or nucleoprotein, and measured secretion of selected cytokines by ELISA. As shown in Figure 3F, there was a slight increase in viral production at 6 hpi, when about 20% of the cells expressed viral antigens and then no more net increase in viral release as up to 80% of the cells expressed viral proteins by 48 hpi. The viral antigen staining was due to viral replication, since there was no signal with UV-inactivated virus (Figure 3E). Despite the abortive release of infectious virus, PR/8 infection induced a time dependent cytokine and chemokine response in human AM (Figure 3G–K). Viruses triggered an early and rapid secretion of IFN-α and CXCL10 at 6 hpi. Secretion of CCL5 and CXCL8 followed the pattern of the viral protein synthesis increasing with time. The virus-induced increase of TNF-α peaked at 24 hpi and then declined. UV-inactivation abolished the virus-stimulated TNF-α production, significantly decreased secretion of IFN-α, CXCL8, and CCL5. However, the inactive virus was able to stimulate a strong CXCL10 response, although the degree was slightly smaller than that from live PR/8 (Figure 3I). The different patterns of the induction suggest that the cytokine response may involve different regulatory mechanisms. In addition, we compared the alterations in mRNA levels of selected innate immune response genes at 3 and 24 hpi for both UV-inactivated PR/8 and live PR/8 infections. Consistent with the protein data, both live and UV-inactivated PR/8 stimulated a large increase in CXCL10 mRNA at both time points. UV-inactivated PR/8 stimulated an up to 4 fold increase of CCL5 and IFNA1. UV-inactivated virus did not alter mRNA levels of RIG-I, TLR7, or ISG56 at either time point (data not shown). These results indicate that viral replication is required for most selected innate immune responses but not required for the CXCL10 response.

### Contemporary influenza virus induces a similar response as PR/8

**Figure 4:**
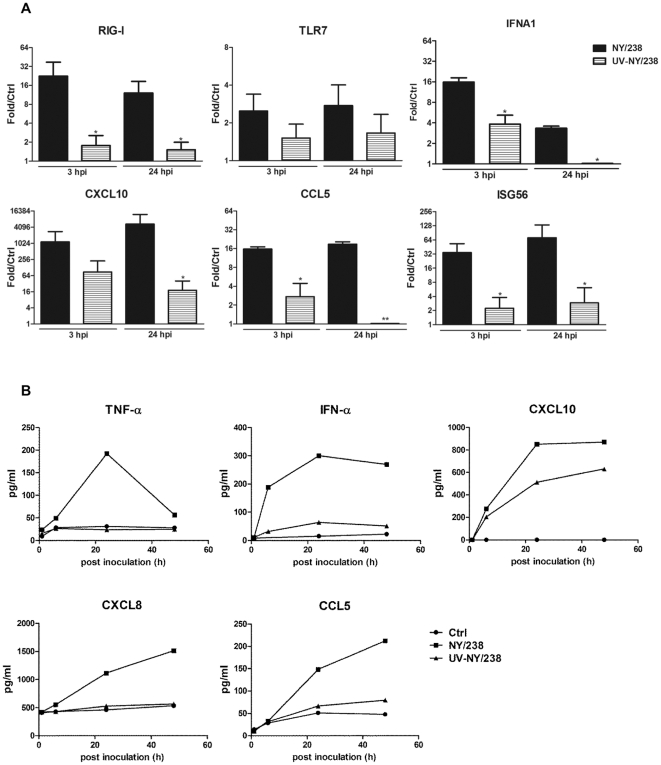
Innate immune response of both live and UV-inactivated contemporary H3N2 influenza viruses-infected AM. Human AM isolated from donor lungs were cultured and infected by live NY/238 virus at a MOI of 0.1 or the equal amount of UV-inactivated NY/238. Cells were harvested at designated times for evaluation of their innate immune response. Panel A. Alterations in mRNAs of innate immune response-related genes at 3 and 24 hpi by realtime RT-PCR. The data represent mean+SE of the relative expression levels of each gene in infected cultures compared to that of non-infected controls after normalization to the level of the constitutive probe cyclophilin B, N = 4. * indicates P<0.05 and ** indicates P<0.01 between live and UV-inactivated cells. Panel B. Kinetics of cytokine and chemokine response by ELISA. The data show representative release of TNF-α, IFN-α, CXCL10, CXCL8, and CCL5 from both live and UV-inactivated NY/238 virus-infected AM from one of 6 donors that all showed similar response.

To investigate whether the results observed with PR/8 can be extended to contemporary human influenza virus infection, we performed a time-course experiment using a H3N2 virus NY/238, a influenza virus rescued by reverse genetics technology based on a swab sample from a patient from New York during the winter of 2005 [23]. Consistent with the results from PR/8 infection, human AM do not support a productive NY/238 infection as verified by no increase in infectious viral particles released from infected culture as measured by plaque assay (data not shown). As shown in Figure 4A, NY/238 infection markedly stimulated CXCL10 mRNA, NY/238 virus also triggered an early increase in the expression of RIG-I and IFNA1 genes and increased mRNA levels of antiviral gene ISG56 and CCL chemokine CCL5. Inoculation with the same amount of UV-inactivated NY/238 virus was able to stimulate an IFNA1 and CXCL10 response. However, the response was smaller than that observed with live virus. Unlike PR/8, NY/238 virus did not induce a significant increase in TLR7 mRNA (Figure 4A). At the protein level, NY/238 virus induced a similar response as PR/8 virus in terms of cytokine and IFN production (Figure 4B). Consistent with the finding with PR/8, viral replication was required for most chemokine and cytokine response and but was not requisite for CXCL10 release.

### Targeting TNF and IL-1 signaling independently reduces the virus-induced secretion of CXCL8 and CCL5

**Figure 5:**
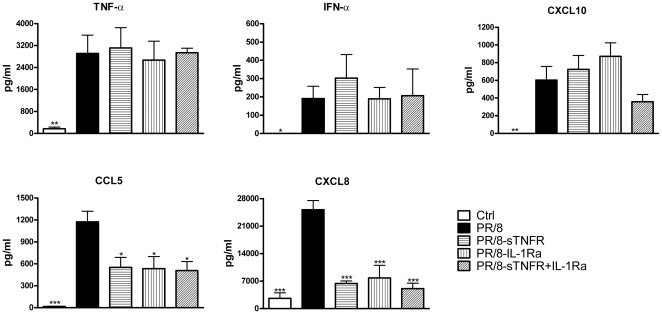
Inhibition of TNF and/or IL-1 pathways decreases release of CXCL8 and CCL5 but not CXCL10 and IFNs induced by influenza infection. Human AM isolated from donor lungs were cultured and infected by PR/8 at a MOI of 0.5. Soluble TNF p55 receptor and IL-1Ra were added to the cultures at 10 µg/ml 45 min before the infection and added back to the cultures after viral inoculation. Secretion of chemokines and cytokines was measured by ELISA at 24 hpi. The data represent mean+SE of each released cytokine and chemokine (pg/ml). N = 6. * indicates P<0.05, ** indicates P<0.01, *** indicates P<0.001 vs. virus-infected cells.

Because PR/8 infection increased secretion of TNF-α and increased gene expression of IL-1 family members, well-known proinflammatory mediators that cause release of inflammatory chemokines, we were interested in the impact of these proinflammatory mediators on the overall chemokine response during the infection in human AM. Our hypothesis was that TNF and IL-1 signaling would augment chemokine secretion in a paracrine manner [29]. As shown in Figure 5, neutralization of TNF pathway by its soluble receptor significantly decreased secretion of CXCL8 by 65% (P<0.001) and CCL5 by 53% (P<0.05), but did not alter secretion of IFNs, CXCL10, or TNF-α itself. Blockade of the IL-1 receptor by its naturally occurring receptor antagonist IL-1Ra [30] had a similar effect. When the activity of both cytokines was inhibited, there was no further reduction in chemokines greater than that of a single inhibitor, although there was a slight decrease in CXCL10 response in the presence of both inhibitors, the response was not statistically significant.

### Influenza infection decreases mRNA expression of macrophage receptor genes and impairs phagocytosis of zymosan

**Figure 6:**
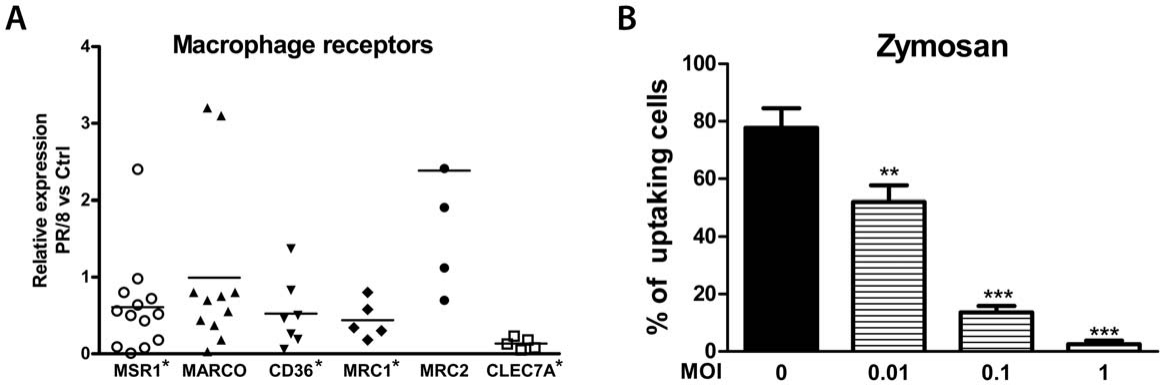
Influenza virus infection decreases CLEC7A (Dectin1) mRNA and reduces phagocytosis of zymosan by AM. Panel A. Human AM were cultured and infected by PR/8 at a MOI of 0.5. The total RNA from infected and non-infected cells was evaluated for the expression of macrophage receptor genes by real-time RT-PCR at 24 hpi. The data show the relative expression levels of each gene in virus-infected cells compared to that of non-infected cells after normalized to the expression of constitutive probe from 4 to 14 donors. Each symbol indicates one donor. * indicates there was a significant difference between control and virus-infected cells (P<0.05). Panel B. PR/8 infection induced a dose-dependent decrease of uptake of zymosan. Isolated AM were cultured and infected by PR/8. At 24 hpi, fluorescent FITC-labeled zymosan was added without serum for 2 h and then the cells were washed and fixed with paraformaldehyde. Uptake of zymosan was measured as percent of cells containing zymosan evaluated under fluorescent microscopy. The data represent mean+SE of percent cells uptaking zymosan. N = 4. ** indicates P<0.01, *** indicates P<0.001 vs. non-infected cells.

AM are important phagocytes and express many scavenger receptors. The microarray experiments indicated that PR/8 infection also significantly decreased mRNA levels of many macrophage receptors especially at 24 hpi (Table 2 and Data S1). We, therefore, investigated the impact of influenza infection on expression of scavenger receptors by real-time RT-PCR. Consistent with the results from microarray experiments, PR/8 infection significantly decreased the mRNA levels of CLEC7A (Dectin 1), macrophage scavenger receptor 1 (MSR1), CD36, and the mannose receptor C type 1 (MRC1) but did not change the expression of MRC2. However, we were not able to confirm the decrease of MARCO, due to the large variation in responses among different donors (Figure 6A). To further investigate if the decrease in macrophage receptor expression was associated with functional consequences, we evaluated the uptake of zymosan, which are yeast walls recognized by CLEC7A, and heat-killed *S. aureus*. As shown in Figure 6B, PR/8 infection reduced uptake of zymosan by AM at 24 hpi in a dose dependent manner. We did not observe a significant cell loss or cytopathic effect at 24 or 48 hpi, although most cells were infected as seen in Figure 3A–C. In addition, PR/8 infection did not affect uptake of heat-killed *S. aureus* until 72 hpi, when the infection induced a significant cytopathic effect (data not shown).

## Discussion

Alveolar macrophages produce a robust innate immune response to influenza. This includes a significant induction of cytokines and chemokines, pathogen recognition, and apoptotic responses, which are similar to the responses of human monocyte derived macrophages [16], [17]. Consistent with other studies of avian or human influenza infections in humans and animals [16], [17], [31]–[33], PR/8 stimulated an early and prominent IFN response in human AM despite of the failure to release infectious viral particles. Human AM produce both type I and type III interferons (Figures 1 and 2). In contrast, alveolar epithelial cells do not produce any type I interferon IFN-α in response to influenza [34]. These results indicate a cell-specific pattern in producing IFN in response to viral infection. It is well known that RIG-I like RNA helicases (RLHs) and TLRs are the two main PRRs responsible for IFN production against RNA viruses including influenza. RLHs (RIG-I and MDA-5) recognize cytoplasmic viral double-stranded RNA, whereas TLRs (TLR3 and TLR7) sense viral nucleic acid in the endosomal compartment [35], [36]. In the current study, PR/8 infection up-regulated mRNA levels of RIG-I and MDA-5 mainly at 4 hpi, but the mRNAs of TLR3 and 7 mainly at 24 hpi (Figure 1B), which suggests that RLHs might be the early sensors and TLRs might be the late sensors for PR/8 in human AM. These results correlate well with those reported by Takeuchi and Thompson that RLHs were responsible for local production of IFNs, whereas TLRs were mainly involved in the late stages of systemic infection [35], [36]. At early times PR/8 triggered mainly pro-inflammatory responses, whereas at later times PR/8 also activated pathways involved in the maintenance of homeostasis such as the activation of IL-10 and IL-6, as well as up-regulation of SOCS genes (Data S1). Therefore, therapeutic regulation of the inflammatory response in acute lung injury should consider both strategies to inhibit secreted cytokines but also strategies to dampen the innate immune response by stimulating IL-10 and SOCS genes. We were able to confirm the results found with PR/8 in contemporary influenza virus NY/238-infected human AM with the exception of an increase in TLR7 mRNA. This might be due to a lower MOI of virus used in the experiments because of the limitation of the viral titer, but it could also be due to differences in the natures of these two viruses or the difference in methods for propagating these two viruses.

CXCL9–11 were the most highly induced chemokines by influenza viruses as verified at both mRNA and protein levels (Figures 1 and 2). These three chemokines bind to a common receptor CXCR3, and the importance of CXCR3 signaling has been shown in the pathogenesis of several viruses including influenza [32], [37]–[39]. CXCL10 is highly induced in avian flu (H5N1)-infected ferrets, non-human primates, and human cells including alveolar epithelial cells and monocyte-derived macrophages [16]–[18], [32], [33], [40], and has been viewed as a prognostic marker for several viral infections [37], [39], [41], [42]. In mice, the peak level of CXCL11 mRNA coincides with the peak of the viremia [43], and the CXCL11 protein has been reported to have anti-viral activity [44]. In addition, all three CXCR3 ligands can induce epithelial cell chemotaxis and proliferation and perhaps accelerate epithelial wound repair during the resolution of viral infections [45], [46]. The robust induction of CXCL9, 10, and 11 in both AM (Figures 1 and 2) and human alveolar type II cells [34] as well as the distinct CXCL10 response induced by both live and UV-inactivated influenza virus PR/8 and contemporary virus NY/238 (Figure 3 and 4) suggest that this family of proteins likely plays an important role in the human lung alveolar defense against influenza infection, which will require further study.

The response of alveolar macrophages was different in a several ways from that reported for human monocyte derived macrophages. The major difference is that alveolar macrophages infected with human influenza viruses do not release much infectious virus, whereas human monocyte-derived macrophages do ([19], [20] and Figure 3). The mechanism for the non-productive infection was not investigated in this study and is likely complicated. One of the possible mechanisms might be related to the lack of gene expression of transmembrane protease serine S1 member 2 (TMPRSS2) and human airway trypsin-like protease (HAT) by human AM (microarray data not shown). Both TMPRSS2 and HAT are type II transmembrane serine proteases [47] possessing trypsin-like activity and are known to be important for cleaving influenza HA required for productive infection [48]. In recent studies Bottcher et al suggest that TMPRSS2 is mainly responsible for cleavage of newly synthesized HA, whereas HAT cleaves both endocytosed and newly synthesized HA [49]. Therefore, lack of these two gene products in human AM may partially explain the lack of released infectious virus by these cells. In addition, both PR/8 and NY/238 viruses induced an early activation of type I IFN, especially IFN-α (Table 1 and Figures 1, 3, and 4). The strong anti-viral property of type I IFN [50] may also contribute to the non-productive infection in these cells. Further studies will be required to understand the mechanism for the failure of release of infectious viral particles by human AM. In addition, inactivation of influenza by UV did not abolish the influenza viruses-stimulated CXCL10 secretion by AM (Figures 3 and 4), which is different from studies with human monocyte-derived macrophages [21], [51] and with human alveolar type II epithelial cells isolated from the same donors ([34] and data not shown). In those studies, release of CXCL10 is totally dependent on viral replication. The mechanism for the distinct CXCL10 response in human AM will require additional and carefully designed studies. The differences between human AM and monocyte-derived macrophages indicate the importance of investigating the response of AM to influenza infection during the initial phases of infection in the lung because AM are main targets for both human and avian influenza viruses [19].

Chemokine and cytokine responses are required for protection of the host against viral infection. However, an exuberant response contributes to the influenza-induced morbidity and mortality, especially in severe pandemic and avian influenza infections [16], [52]. In the current study, PR/8 infection induced an increase in TNF-α and IL-1β, well-known paracrine proinflammatory factors. Therefore, we hypothesized that inhibiting these factors might reduce the influenza-induced-inflammatory response. Since the contemporary virus NY/238 induced a similar cytokine and chemokine response as PR/8, it would be reasonable to expect that the regulation of chemokine and cytokine in contemporary influenza infection might also be similar to PR/8 infection. As shown in Figure 5, inhibiting TNF and/or IL-1 decreased more than 50% of the PR/8-induced secretion of inflammatory chemokines CXCL8 and CCL5 but did not truly affect type I interferon or CXCL10 response, although we observed a decrease of CXCL10 in the presence of both inhibitors (Figure 5). TNF and IL-1 signaling are known to be regulated by NF-κB and there are several NF-κB binding sites in the promoter of CXCL10 [53], despite of the fact that CXCL10 is an IFN-induced protein [24]. This may explain why inhibiting both pathways slightly decreased the amount of CXCL10 from infected AM. Our results suggest that short term targeting the critical paracrine factors might be beneficial for controlling the excessive infiltration of inflammatory cells and acute lung injury during pandemic or avian flu infection *in vivo*. Of course, this would require careful consideration of time and dose so as not to increase secondary bacterial infections.

Influenza infection significantly decreased mRNA level of macrophage receptors CLEC7A, MSR1, CD36, and MRC1 (Figure 6A and Table 2). CLEC7A belongs to the C-type lectin family and functions as a PRR that recognizes a variety of beta-1, 3-linked and beta-1, 6-linked glucans from fungi. A decrease of CLEC7A in infected AM suggests that these cells might not efficiently recognize and engulf fungi after influenza infection. As shown in Figure 6B, the uptake of zymosan, a yeast cell wall component containing beta-1-3-glycosolic linkeages, was decreased in a dose-dependent manner in PR/8-infected human AM. This effect was not associated with cell loss or cytopathic effect because we did not observe a significant cytopathic effect (Figure 3B) even at a MOI of 1 (data not shown). However, the explanation of the decreased uptake might be more complicated than simply the loss of this receptor. In addition, other macrophage receptors MSR1, MARCO, CD36, as well as mannose receptor MRC1 are important for bacterial and particle uptake [54]–[56]. Mice with deletions of MSR1 or CD36 have increased susceptibility to pneumococcal or staphylococcal pneumonia [57]–[59]. Although impairment of macrophage phagocytosis of bacteria after influenza in mice is well recognized [60], [61] and secondary bacterial infection after influenza is a common clinical problem, we were not able to detect a significant decrease in uptake of heat-inactivated *S. aureus* in human AM until 72 hpi, at which time the cytopathic effect was significant. We did not observe a consistent decrease of MSR1 protein by flow cytometry in PR/8-infected AM, which might explain why the infection did not impair the bacterial uptake (data not shown). We were also not able to verify the decrease of mRNA level of MARCO, another important macrophage scavenger receptor for influenza infections in mice and human cells [54], [56], [58], [62]. Nine of 11 donors showed a decrease in mRNA levels of MARCO after infection with PR/8 (Figure 6A). Two other donors had an increase in levels of MARCO mRNA. Therefore, changes of bacteria-related receptors in human AM after influenza require additional studies, and there may be variations in response among individuals.

In summary, we performed a global profiling of innate immune response and regulation with a focus on chemokine and cytokine response in influenza-infected human AM. Human AM are apparently different from human monocyte derived macrophages in their ability to release infectious virus and the CXCL10 response to UV inactivated virus. Future studies should compare these responses in peripheral and alveolar macrophages from the same donors. In addition, during acute lung injury, short term targeting of paracrine inflammatory factors such as TNF and IL-1 as well as targeting IL-10 and SOCS genes might decrease the acute injury and allow for better gas exchange.

## Methods

### Isolation and culture of human alveolar macrophages

AM were isolated from deidentified human donor lungs, which were not suitable for transplantation and donated for medical research. We obtained the donor lungs through the International Institute for the Advancement of Medicine (Edison, NJ) and the National Disease Research Interchange (Philadelphia, PA) [3]. The Committee for the Protection of Human Subjects at National Jewish Health approved this research and has designated this research as non-human project. The isolated AM could be frozen and recovered in 90% FBS and 10% DMSO. There was no apparent difference in response with frozen or freshly isolated macrophages in terms of the level of infection and virus-induced TNF-α secretion (data not shown). AM were plated in DMEM/10% FBS with antibiotics, and cultured at 37°C in 10% CO_2_ overnight. The cells were then washed and cultured for another day in DMEM and 1% charcoal stripped FBS with antibiotics before viral infection. Their purity was measured by staining for CD68 (Dakocytomation, Carpinteria, CA) [3].

### Virus preparation and infection

Influenza A virus A/PR/8/34 (PR/8) was grown in 10-day-old SPF Premium Eggs (Charles River SPAFAS. North Franklin, CT) and prepared as described previously [3]. Contemporary influenza H3N2 virus, A/New York/238/2005 (NY/238), was created by reverse genetics using plasmids that corresponded to the consensuses sequence obtained from a human swab specimen collected in New York State in the winter of 2005 [23]. NY/238 was passaged in Madin-Darby Canine Kidney (MDCK) cells and the viral titer was measured by plaque assay on MDCK cells as described previously [34]. Briefly, stocks of purified virus was serially diluted in DMEM with 1 µg/ml TPCK trypsin (Sigma-Aldrich, St. Louis, MO) and used to inoculate triplicate wells of near confluent MDCK cells. After a 1 h inoculation, the inoculum was removed and the cells were overlaid with MEM with 4% FBS and 0.5% SeaKem LE agarose (Cambrex, Rockland, ME). Plaques were stained and counted after 72 h incubation at 37°C, with the agarose overlay medium containing 10% neutral red (Sigma-Aldrich). For UV-inactivation of PR/8 or NY/238, 500 µl diluted virus was placed in a 35-mm^2^ petri dish on ice and irradiated twice in a UV Stratalinker (Stratagene, La Jolla, CA) at a cumulative dose of 120 mJ/cm^2^. Viral inactivation was demonstrated by plaque assay on MDCK cells as described above.

On the day of infection, AM were inoculated with live PR/8 at a designated multiplicity of infection (MOI) or with the same amount of UV-inactivated PR/8 for 1 h. After inoculation, cells were washed and then cultured until harvest. Influenza infection was verified by immuno-fluorescent staining with goat antibody to the hemagglutinin of PR/8 (kindly provided by BEI Resources, Manassas, VA). For NY/238 infection, AM was inoculated with a MOI of 0.1 instead of 0.5 due to the limitation of viral titer and infection was confirmed by immuno-fluorescent staining with mouse antibody to influenza nucleoprotein (Millipore, Billerica, MA).

For the inhibition experiments, cells were treated with 10 µg/ml human IL-1 receptor antagonist (IL-1Ra) [63] and extracellular TNF neutralization was achieved by treating cells with 10 µg/ml recombinant human soluble TNF receptor (sTNFR) [64]. Both IL-1Ra and/or sTNFR were added to the cells for 45 min before virus inoculation. DMEM alone was used as vehicle control for both inhibitors. After inoculation, cells were washed and cultured with the inhibitors for an additional 24 h.

### Affymetrix microarray experiments

At 4 and 24 hpi, total RNA from virus-infected and non-infected AM from three donors was extracted and purified using RNeasy kit (QIAGEN, Valencia, CA). The samples were run on Affymetrix HG-U133 Plus 2.0 chips (Affymetrix, Santa Clara, CA) and processed as indicated by the manufacturer in the Microarray Core of the University of Colorado Denver. All data is MIAME compliant and the raw data had been deposited in a MIAME compliant database Gene Express Omnibus (GEO). The GEO accession numbers are GSM762686, GSM762687, GSM762688, GSM762689, GSM762694, GSM762695, GSM762696, GSM762697, GSM762702, GSM762703, GSM762704, GSM762705. Analyses of microarray data were performed using R statistical package from Bioconductor open source software for bioinformatics. Prior to statistical analyses, raw data from array scans were processed using the Robust Multi-chip Average (RMA) normalization method to subtract a background value [34]. After normalization, data were filtered to exclude all probe sets with an “absent” call in all samples and to remove transcripts that demonstrated little variation across all arrays by comparing the variances of the log-intensities for each gene with the median of all variances for the entire array. The filtered gene list was generated using the Student's T test to select statistically significant genes and corrected using the False Discovery Rate approach. Genes that had at least a 2-fold change in comparison to the uninfected controls for all three subjects were used for further analyses.

### Real-time RT-PCR

mRNA expression of selected genes that were significantly up-regulated by PR/8 or NY/238 were validated by quantitative real-time RT-PCR [34]. These genes include IFNs, PRRs, chemokines, and SOCSs. Except for IFN-β and IL-29 genes whose probes were synthesized in house [34], the specific probes for other genes were purchased from Applied Biosystems (Applied Biosystems Inc. Foster City, CA). The expression level of each specific gene was normalized to the level of a constitutive probe cyclophilin B [34].

### Enzyme linked immunosorbent assay (ELISA)

Supernatant from PR/8 OR ny/238-infected and non-infected cells were harvested at designated times after inoculation for the measurement of chemokine and cytokine secretion by ELISA. The ELISA kits for human CXCL9, CXCL10, CXCL11, CCL5, CXCL8, and IL-29 were purchased from ELISA Tech (ELISA Tech, Aurora, CO). The ELISA kit for IFN-α was purchased from Invitrogen (Invitrogen, Carlsbad, CA).

### Uptake of zymosan and heat killed *S. aureus*

Human AM were cultured and infected with PR/8 at the designated MOI. Uptake of zymosan or heat-killed *S. aureus* were performed according to manufacturer's instructions. For uptake of zymosan, cells were incubated with fluorescent-labeled zymosan A Bioparticles (Invitrogen) at a ratio of 10 particles per cell for 2 h, then cells were washed and fixed with 4% paraformaldehyde for 10 min. The uptake was analyzed by fluorescent microscopy. For uptake of heat-killed *S. aureus*, cells were incubated with pHrodo-labeled, heat-killed *S. aureus* (pHrodo-SA) (Invitrogen) at a ratio of 20 particles per cell for 2 h. The cells were then washed to remove non-internalized particles, collected, and fixed with 4% paraformaldehyde. Uptake of the pHrodo-SA was analyzed on the LSR II flow cytometer (BD Biosciences) in the National Jewish Health Flow Cytometry Core, and the data were analyzed using FlowJo software (TreeStar, Ashland, OR). In addition to the PR/8 infected cells, positive control uninfected cells and negative control paraformaldehyde fixed cells were also used.

### Statistics

Statistical analyses were conducted in GraphPad Prism version 5.0 (GraphPad Software, San Diego, CA). Pair-wise comparisons were tested for significance using Wilcoxon matched pairs test or Paired T test. Comparison among three or more groups was performed using one-way ANOVA with Tukey's post test analysis.

## Supporting Information

Data S1 Human AM from 3 non-smoking donors were isolated, cultured, and infected by PR/8 virus at MOI of 0.5. The gene profiling of infected and non-infected cells at 4 and 24 hpi from each donor was examined by microarray experiments using Affymetrix HG-U133 Plus 2.0 chips (Affymetrix, Santa Clara, CA). The filtered gene list was generated as described in the Section of Methods and Materials. The data show probe ID, gene symbol, gene name, and fold change at 4 and 24 hpi. Red indicates similar results from multiple probes for the same gene, and the probe ID is the representative probe ID from several probes.
(XLS)
